# Differential Regulation and Recovery of Intracellular Ca^2+^ in Cerebral and Small Mesenteric Arterial Smooth Muscle Cells of Simulated Microgravity Rat

**DOI:** 10.1371/journal.pone.0019775

**Published:** 2011-05-18

**Authors:** Jun-Hui Xue, Lian-Hong Chen, Hua-Zhou Zhao, Yong-Dong Pu, Han-Zhong Feng, Yu-Guang Ma, Jin Ma, Yao-Ming Chang, Zuo-Ming Zhang, Man-Jiang Xie

**Affiliations:** 1 Department of Aerospace Clinical Medicine, Key Laboratory of Aerospace Medicine of Ministry of Education, Fourth Military Medical University, Xi'an, Shaanxi Province, China; 2 Department of Chest Surgery, Tangdu Hospital of Fourth Military Medical University, Xi'an, Shaanxi Province, China; 3 309 Clinical Divisions, Department of General Surgery, General Hospital of PLA, Beijing, China; 4 Department of Aerospace Physiology, Key Laboratory of Aerospace Medicine of Ministry of Education, Fourth Military Medical University, Xi'an, Shaanxi Province, China; Istituto Dermopatico dell'Immacolata, Italy

## Abstract

**Background:**

The differential adaptations of cerebrovasculature and small mesenteric arteries could be one of critical factors in postspaceflight orthostatic intolerance, but the cellular mechanisms remain unknown. We hypothesize that there is a differential regulation of intracellular Ca^2+^ determined by the alterations in the functions of plasma membrane Ca_L_ channels and ryanodine-sensitive Ca^2+^ releases from sarcoplasmic reticulum (SR) in cerebral and small mesenteric vascular smooth muscle cells (VSMCs) of simulated microgravity rats, respectively.

**Methodology/Principal Findings:**

Sprague-Dawley rats were subjected to 28-day hindlimb unweighting to simulate microgravity. In addition, tail-suspended rats were submitted to a recovery period of 3 or 7 days after removal of suspension. The function of Ca_L_ channels was evaluated by patch clamp and Western blotting. The function of ryanodine-sensitive Ca^2+^ releases in response to caffeine were assessed by a laser confocal microscope. Our results indicated that simulated microgravity increased the functions of Ca_L_ channels and ryanodine-sensitive Ca^2+^ releases in cerebral VSMCs, whereas, simulated microgravity decreased the functions of Ca_L_ channels and ryanodine-sensitive Ca^2+^ releases in small mesenteric VSMCs. In addition, 3- or 7-day recovery after removal of suspension could restore the functions of Ca_L_ channels and ryanodine-sensitive Ca^2+^ releases to their control levels in cerebral and small mesenteric VSMCs, respectively.

**Conclusions:**

The differential regulation of Ca_L_ channels and ryanodine-sensitive Ca^2+^ releases in cerebral and small mesenteric VSMCs may be responsible for the differential regulation of intracellular Ca^2+^, which leads to the altered autoregulation of cerebral vasculature and the inability to adequately elevate peripheral vascular resistance in postspaceflight orthostatic intolerance.

## Introduction

Postspaceflight orthostatic intolerance has been regarded as one of the major adverse effects of microgravity exposure and there are still no effective countermeasures [1], [2]. Human studies from spaceflight research and bed rest have indicated that the altered autoregulation of cerebral vasculature and the inability to adequately elevate peripheral vascular resistance may be the fundamental causes in the occurrence of orthostatic intolerance after spaceflight [3], [4]. In the past decades, ground-based animal studies with tail-suspended hindlimb-unweighting rat model, which has been widely used to simulate physiological effects of microgravity [5], have revealed that simulated microgravity induced cerebrovascular adaptations including the increased myogenic tone, enhanced vasoreactivity, hypertrophic remodeling, and endothelial dysfunction [6], [7], whereas, simulated microgravity induced small mesenteric arterial adaptations including the decreased myogenic tone, attenuated vasoreactivity, atrophic remodeling, and endothelial dysfunction [7], [8]. These findings suggest that differential adaptations of cerebrovasculature and small mesenteric arteries could be one of critical factors in postspaceflight orthostatic intolerance, but the cellular mechanisms remain unknown.

Intracellular Ca^2+^ in vascular smooth muscle cells (VSMCs) is an important determinant for functional and structural adaptations in vasculature [9]. Ca^2+^ influx from the long-lasting voltage-dependent Ca^2+^ (L-type, Ca_L_) channels in plasma membrane and Ca^2+^ releases from the ryanodine receptors (RyRs) in sarcoplasmic reticulum (SR) are likely to play the essential roles in controlling intracellular Ca^2+^ [10]. It is known that the increased intraluminal pressure depolarizes VSMCs and then enhances the extracellular Ca^2+^ influx by opening Ca_L_ channels. The increased concentration of intracellular Ca^2+^ ([Ca^2+^]_i_) subsequently activates RyRs and produces the transient local Ca^2+^ release events in micromolar concentrations (Ca^2+^ sparks) from SR, which in turn activate nearby Ca^2+^-activated K^+^ (K_Ca_) channels in plasma membrane, leading to membrane hyperpolarization, inhibition of Ca_L_ channels, and thereby favoring vasodilation by reducing the Ca^2+^ influx [11]. Therefore, Ca_L_ channels in plasma membrane and RyRs in SR are important mediators to control arterial excitation-contraction coupling and subsequent structural remodeling by handling intracellular Ca^2+^. It has been demonstrated that hypertension [12], atherosclerosis [13], diabetes [14], and hypoxia [15] are all associated with the abnormal function of Ca_L_ channels or RyRs.

Our previous work reported that 28-day simulated microgravity increased the concentration of intracellular Ca^2+^ in rat cerebral VSMCs [16] associated with the upregulation of Ca_L_ channels [17]. In contrast, there is a report that 14-day hindlimb unloading decreased the level of intracellular Ca^2+^ in rat small mesenteric VSMCs by reducing the function of ryanodine-sensitive Ca^2+^ releases associated with the downregulation of RyR2 mRNA and protein expression [18]. We also reported that 28-day simulated microgravity down-regulated the Ca_L_ channels in rat small mesenteric VSMCs [17]. These results suggested that alterations of Ca_L_ channels in plasma membrane and ryanodine-sensitive Ca^2+^ releases from SR may account for the changes of intracellular Ca^2+^ in cerebral and small mesenteric VSMCs of simulated microgravity rats. Furthermore, these results also indicated that Ca_L_ channels and ryanodine-sensitive Ca^2+^ releases may play an important role in the differential adaptations of cerebral and small mesenteric arteries in simulated microgravity rats. However, it is not clear whether the alterations of Ca_L_ channels in plasma membrane are accompanied by the changes of ryanodine-sensitive Ca^2+^ releases from SR in cerebral or small mesenteric VSMCs of simulated microgravity rats, respectively. In addition, it is not clear whether the function of Ca_L_ channels and ryanodine-sensitive Ca^2+^ releases could recover after removal of suspension. The purpose of the present work was 1) to investigate the function of Ca_L_ channels by comparing the whole-cell currents, protein expressions of α_1C_-subunit in cerebral and small mesenteric VSMCs isolated from control rats, simulated microgravity rats, and rats after removal of suspension, and 2) to assess the functions of ryanodine-sensitive Ca^2+^ releases by measurements of intracellular Ca^2+^ in response to caffeine in cerebral and mesenteric VSMCs isolated from the same groups as described above. Taken together, the present study provided initial evidences that there is a differential regulation and recovery in the functions of Ca_L_ channels and ryanodine-sensitive Ca^2+^ releases of cerebral and small mesenteric VSMCs from simulated microgravity rats, respectively, which may provide a novel mechanism for cerebral and small mesenteric arterial adaptations during microgravity.

## Materials and Methods

All animal procedures described in this study were performed in adherence with *the Guide for the Care and Use of Laboratory Animals* published by the US National Institutes of Health (NIH Publication No. 85-23, revised 1996), with approval from Committee on the Ethics of Animal Experiments of the University of Fourth Military Medical University (Permit Number: 10001). All surgery was performed under sodium pentobarbital anesthesia, and all efforts were made to minimize suffering. Unless otherwise stated, all chemicals and reagents used in this study were obtained from Sigma Chemical Company (St. Louis, Missouri, USA).

### Animal model

The tail-suspended, hindlimb-unweighting rat model, which was described in detail previously [5], was used to simulate the cardiovascular effects of microgravity. The present study was divided into two experiments. *Experiment I* was designed to investigate the functions of Ca_L_ channels and ryanodine-sensitive Ca^2+^ releases in cerebral and small mesenteric VSMCs isolated from control rats, simulated microgravity rats, and tail-suspended rats submitted to a recovery period of 3 days after removal of suspension. *Experiment II* was designed to investigate the function of Ca_L_ channels in small mesenteric VSMCs isolated from simultaneous control rats and tail-suspended rats submitted to a recovery period of 7 days after removal of suspension. In *Experiment I*, a total of 90 male Sprague-Dawley rats, 7–9 weeks of age and weighing ∼200 g, were randomly assigned into 3 groups (*n* = 30/group): 28-day simultaneous control (CON-28 d), 28-day tail-suspension (SUS-28 d), and tail-suspended rats submitted to a recovery period of 3 days at the end of 28-day suspension (SUS+Rec-3 d). In *Experiment II*, 20 male Sprague-Dawley rats, 7–9 weeks of age and weighing ∼200 g, were randomly assigned into 2 groups (*n* = 10/group): 35-day simultaneous control (CON) and tail-suspended rats submitted to a recovery period of 7 days at the end of 28-day suspension (SUS+Rec-7 d). The SUS rats were maintained in an about −30° head-down tilt position with their hindlimbs unloaded and caged individually in a room maintained at 23°C on a 12∶12-h light-dark cycle. The CON rats and rats submitted to a recovery period were maintained in individual cages and were treated similarly except for the tail suspension. All the animals received standard lab chow and water ad libitum. Animals were anesthetized with pentobarbital sodium (50 mg/kg ip) and killed by exsanguinations via the abdominal aorta. The wet weight of left soleus and the length of left femur were measured to confirm the efficacy of deconditioning and monitor any effect on growth.

### Isolation of VSMCs

Enzymatic isolation of single VSMC was carried out as previously described [17], [19]. Briefly, brain and superior mesenteric tissues were removed rapidly and placed in 4°C physiological salt solution (PSS). PSS contained (in mM) 137 NaCl, 5.6 KCl, 1 MgCl_2_, 0.42 Na_2_HPO_4_, 0.44 NaH_2_PO_4_, 4.2 NaHCO_3_, and 10 HEPES, equilibrated with 95% O_2_ and 5% CO_2_ at pH adjusted to 7.4 with NaOH. The cerebral arteries (superior, middle, and basilar arteries with the circle of Willis) and small mesenteric arteries with its branches were dissected and cut into 1–2 mm length. The cerebral arterial segments were digested for 18 min (small mesenteric arterial segments for 25 min) at 37°C with the solution contained 4 mg/ml papain (Biochrom, Berlin, Germany), 2 mg/ml dithioerythritol (Amresco, St. Louis, Missouri, USA), 1 mg/ml bovien serum albumin (BSA), and 5 mM taurine in PSS. Vessel segments were then transferred to enzyme-free PSS containing 1 mg/ml BSA and 5 mM taurine at room temperature for 10 min and triturated with a flame-polished pipette to disperse VSMCs. Isolated VSMCs were suspended in Ca^2+^-free PSS containing 1 mg/ml BSA and 5 mM taurine and stored at 4°C for use within 8 h.

### Electrophysiological measurements

Patch-clamp recordings were performed as previously described [16], [17], [20]. Cell currents were recorded with an amplifier (CEZ-2300, Nihon Kohden Co., Tokyo, Japan) and a version interface (Axon Instruments, Foster City, California, USA). Command-voltage protocols and data acquisition were performed with pCLAMP software (version 8.0, Axon Instruments). Patch pipettes (tip resistance 2–6 MΩ when filled with a pipette solution) were fabricated on an electrode puller (Narishige Instruments, Tokyo, Japan) with borosilicate glass capillary tubing. A coverslip containing the cells was positioned in a 2-ml recording chamber and superfused with the extracellular (bath) solution. All measurements were performed at room temperature (22–24°C). Resting membrane potential (Em) was measured with the current-clamp configuration of the patch-clamp technique while the cell was held at zero membrane current [21]. The external (bath) solution contained (in mM) 135 NaCl, 4 KCl, 1 MgCl_2_, 2 CaCl_2_, 10 HEPES, and 10 glucose, equilibrated with 95% O_2_ and 5% CO_2_ at pH 7.4 adjusted with NaOH. The pipette solution contained (in mM) 143 KCl, 1 CaCl_2_, 1 MgCl_2_, 3 EGTA, and 10 HEPES, equilibrated with 95% O_2_ and 5% CO_2_ at pH 7.2 titrated with KOH.

Whole-cell Ca_L_ channel currents were measured with the conventional voltage clamp configuration. Cell capacitance (Cm) and access resistance (Ra) were estimated from the capacitive current transient evoked by applying a 20-mV pulse for 40 ms from a holding potential of −60 mV to −40 mV. The cell was held at −40 mV and then stepped in 10-mV increments from −30 to +60 mV. Voltage steps were 250 ms in a duration and 2-s intervals were allowed between steps. Currents were filtered at 0.5 kHz and digitized at 4 kHz. Nonspecific membrane leakage and residual capacitive currents were subtracted using the P/4 protocol. Currents were sampled and averaged while the current amplitude was stabilized. Barium (Ba^2+^) was used rather than Ca^2+^ as the charge carrier to increase unitary currents and to minimize Ca^2+^-dependent run-down. Currents were normalized to Cm to obtain the current densities. To obtain the *I–V* curve of Ca_L_, the current densities were plotted against the corresponding command potentials. Two kinds of external solutions were used, i.e., *solution A* and *B*. *Solution A* was used while making a gigaohm seal between the recording pipette and cell surface. It contained (in mM) 130 NaCl, 5.4 KCl, 1 MgCl_2_, 10 BaCl_2_, 10 HEPES, and 10 glucose, equilibrated with 95% O_2_ and 5% CO_2_ at pH 7.4 adjusted with NaOH. After a seal of 2 GΩ was obtained, the perfusion fluid was changed to *solution B* before current recording. It contained (in mM) 75 Tris-Cl, 50 BaCl_2_, 10 HEPES, and 10 glucose, equilibrated with 95% O_2_ and 5% CO_2_ at pH 7.4 titrated with Tris base. The pipette solution contained 150 CsCl, 1 MgCl_2_, 10 EGTA, 5 HEPES, 5 Na_2_ATP, and 5 Na_2_ creatine phosphate, equilibrated with 95% O_2_ and 5% CO_2_ at pH 7.2 titrated with CsOH. In the present study, extracellular application of 5 µM Bay K 8644 (the specific agonist of Ca_L_) and 0.1 µM nifedipine (the specific blocker of Ca_L_) were used to identify the properties of Ca_L_ as describe before [16], [17].

### Evaluation of Ca_L_ channel protein expression by Western blotting

Protein samples of cerebral and small mesenteric arteries were prepared according to the published methods [17]. Briefly, cerebral and small mesenteric arterial specimens were minced into small pieces and homogenized on ice containing tissue protein extraction reagent (T-PER, Pierce, Rockford, Illinois, USA) and protease inhibitor (Halt, Pierce, Rockford, Illinois, USA). Large tissue debris and nuclear fragments were removed by two centrifuge spins (1,000 rpm for 5 min, 12,000 rpm for 15 min) at 4°C and supernatants were obtained. The concentration of protein samples was determined by the bicinchoninic acid method (Pierce, Rockford, Illinois, USA) using BSA as a standard. Equivalent amounts of proteins from different groups were loaded to adjacent lanes for SDS-PAGE and each sample based on tissue pooled from 4∼5 animals. Protein samples were run for 80 min at 30 mA for electrophoretically size-separation using a 8% Tris-Glycine gel (Invitrogen, Carlsbad, California, USA). After size separation, proteins were transferred onto a nitrocellulose membrane at 100 mA for 3 h and blocked with 5% nonfat dry milk in PBS containing 0.1% (w/v) Tween 20 (PBS-T) overnight at 4°C. Subsequently, the membranes were incubated for 3 h with a 1∶200 dilution of rabbit polyclonal antibody against amino acids 848–865, which corresponds to the C-terminus site of Ca_L_ channel α_1C_-subunit (Alomone Labs, Jerusalem, Israel). The membrane then incubated for 45 min with Infrared (IR)-labeled secondary antibodies (LI-COR) in PBS-T containing 0.01% SDS. A monoclonal mouse antibody raised against the structural protein β-actin (Sigma) was used as a lane-loading control. The bound antibody was detected by the Odyssey infrared imaging system (LI-COR), and the densities of the immunoreactive doublet bands at 200 and 240 kD associated with anti-α_1C_ subunit of Ca_L_ channel were expressed as a percentage of the β-actin density for each lane. Densitometry analysis of bands was performed by Scion image (Scion, Frederick, MD).

### Measurement of intracellular Ca^2+^

In an attempt to establish whether simulated microgravity affects the function of ryanodine-sensitive Ca^2+^ releases from SR in cerebral and small mesenteric VSMCs, respectively, we investigated the average changes of intracellular Ca^2+^ fluorescence intensity in response to caffeine. The intracellular Ca^2+^ was measured with Ca^2+^ indicator, Fluo-3-acetoxymethyl ester (Fluo-3/AM, Molecular Probes, Oregon, USA), as previous described [22]. Fresh cerebral and small mesenteric VSMCs were incubated with Fluo-3/AM in a final concentration of 5 µM for 30 min at 37°C. After incubation, the Fluo-3/AM-loaded cells on coverslips were washed with the Ca^2+^-free balanced salt solution (BSS) in the following composition (mM): 126 NaCl, 5 KCl, 0.3 NaH_2_PO_4_, 10 HEPES, 1 MgCl_2_, 10 glucose, 1 EGTA, equilibrated with 95% O_2_ and 5% CO_2_ at pH 7.4 adjusted with NaOH [18], [23]. The cells were scanned under a laser confocal microscope (Olympus FV1000, Tokyo, Japan) by illuminating with a krypton/argon laser at 488 nm emitted light and capturing the emitting fluorescence at 526 nm. To ensure efficient quantum capture, the cells were placed on the bottom of a recording chamber and images were recorded after 10∼20 s when fluorescence intensity became stable. During continuously scanning, 10 mM caffeine in Ca^2+^-free BSS was administrated to the cell and a period of 3 min was recorded. To avoid any laser-induced change in Ca^2+^ signaling, each cell was scanned only once. Also, only one cell was scanned in each scanning time to avoid cell density-induced changes in fluorescence intensity. The average fluorescence intensity was used to indicate the changes of intracellular Ca^2+^ and the maximal increase of Ca^2+^ fluorescence intensity was used to indicate the function of ryanodine-sensitive Ca^2+^ releases from SR in VSMCs as describe before [24].

### Statistical Analysis

Except the data of body weight are given as means ± SD, all other data are expressed as means ± SE. One-way ANOVA was used to determine the differences of Ca_L_ channel current densities in different groups, followed by a S-N-K-*Post Hoc*. Student's *t*-test was used to determine significant differences of body weights, soleus weights, femur lengths, and the maximal increases of Ca^2+^ fluorescence intensity in different groups. A value of *P*≤0.05 was considered to be statistically significant.

## Results

### Physical characteristics of experimental animals

As summarized in Table 1, there were no significant differences in either final body weights or the lengths of left femur among CON, SUS, and SUS+Rec groups in *Experiment I* and *Experiment II*, suggesting a normal growth rate during and after simulated microgravity. However, after 28-day tail-suspension, the wet weights of left soleus in SUS-28 d rats were about 58.7% less than that in CON-28 d rats, indicating the deconditioning effects of simulated microgravity. In addition, the wet weights of left soleus in SUS+Rec-3 d rats were 24.3% less than that in CON-28 d rats, which means 3-day recovery after removal of 28-day tail-suspension could only partially recover the mass reduction of atrophic soleus due to simulated microgravity. There were no significant difference in the wet weights of left soleus between CON-35 d and SUS+Rec-7 d rats, suggesting a 7-day recovery after removal of 28-day tail-suspension could completely recover the mass reduction of soleus.

**Table 1:** Body weight, the length of left femur, and the wet weight of left soleus in CON, SUS, and SUS+Rec rats.

	Body weight (g)		Length of left femur (mm)	Wet weight of left soleus (mg)
	Initial	Final		
*Experiment I*				
CON-28 d (n = 30)	229.3±7.5	358.8±8.5	35.6±0.8	150.8±2.4
SUS-28 d (n = 30)	228.6±8.1	361.3±9.2	35.4±0.6	62.3±2.1**
SUS+Rec-3 d (n = 30)	227.5±8.8	372.8±9.9	35.7±0.5	114.2±2.5**
*Experiment II*				
CON-35 d (n = 10)	218.7±13.6	403.8±14.5	38.5±1.3	155.0±4.2
SUS+Rec-7 d (n = 10)	226.7±12.3	396.3±13.1	37.3±0.9	148.0±2.2

Values of body weights are means ± SD; others are means ± SE. CON-28 d: 28-day control rats; SUS-28 d: 28-day tail-suspended rats, SUS+Rec-3 d: tail-suspended rats submitted to a recovery period of 3 days at the end of 28-day suspension. CON-35 d: 35-day control rats; SUS+Rec-7 d: tail-suspended rats submitted to a recovery period of 7 days at the end of 28-day suspension.

**P<0.01 *vs.* CON.

### Simulated microgravity depolarized the cerebral VSMCs and hyperpolarized the small mesenteric VSMCs

**Figure 1:**
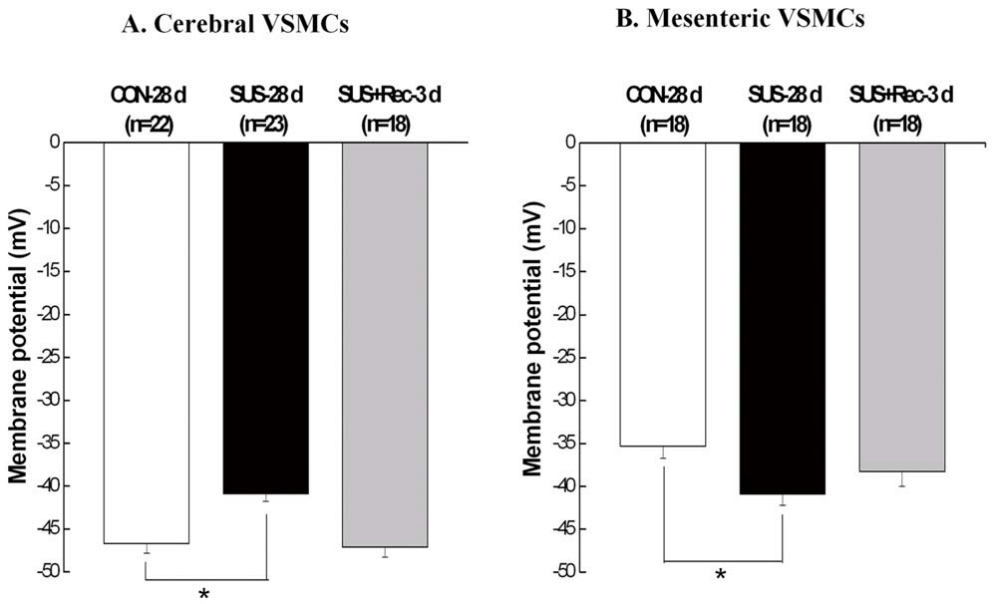
Comparisons of membrane potential in cerebral (A) and small mesenteric VSMCs (B) isolated from CON-28 d, SUS-28 d, and SUS+Rec-3 d rats. CON-28 d: 28-day simultaneous control rats; SUS-28 d: 28-day tail-suspended rats, SUS+Rec-3 d: tail-suspended rats submitted to a recovery period of 3 days at the end of 28-day suspension. Values are means ± SE with the number of cells recorded in parentheses. *P<0.05 *vs.* CON.

For cerebral VSMCs, cell capacitance (Cm), an index of cell membrane area, did not show significant differences among CON-28 d (23.2±0.6 pF, n = 22), SUS-28 d (22.8±0.6 pF, n = 17), and SUS+Rec-3 d rats (23.6±0.9 pF, n = 26). Correspondly, there were neither significant changes in Cm of small mesenteric VSMCs among CON-28 d (23.4±0.9 pF, n = 24), SUS-28 d (23.1±0.6 pF, n = 23), and SUS+Rec-3d rats (23.8±0.7 pF, n = 23). These results suggested that simulated microgravity did not affect the cell surface areas of cerebral and small mesenteric VSMCs, which were consistent with our previous report [16], [21]. As compared with that in CON-28 d rats, 28-day tail-suspension significantly depolarized the membrane potential of cerebral VSMCs (Fig. 1A) and significantly hyperpolarized the membrane potential of small mesenteric VSMCs (Fig. 1B). However, 3-day recovery after removal of suspension could completely recover the membrane potential to their control levels in cerebral and small mesenteric VSMCs, respectively (Fig. 1). These results suggested simulated microgravity induced a differential regulation of membrane potential in cerebral and small mesenteric VSMCs, respectively, which are in general agreement with previous report [16], [21].

### Simulated microgravity increased the current densities of Ca_L_ channel in cerebral VSMCs and decreased the current densities of Ca_L_ channel in small mesenteric VSMCs

**Figure 2:**
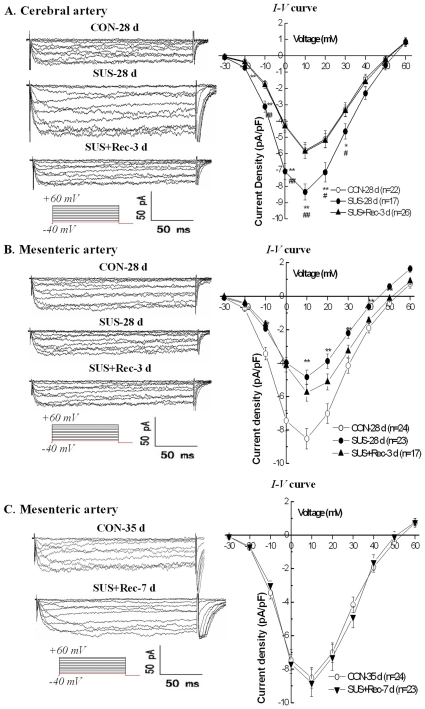
Comparison of whole-cell Ca_L_ current densities in cerebral (A) and small mesenteric VSMCs (B and C) isolated from CON, SUS, and SUS+Rec rats. CON: simultaneous control rats, SUS: 28-day tail-suspended rats, SUS+Rec: tail-suspended rats submitted to a recovery period of 3 or 7 days at the end of 28-day suspension. Representative recordings in the left panel were used to show the whole-cell Ca_L_ currents in different groups. The mean *I–V* curves were further expressed in terms of current densities in the right panel. Values are means ± SE with the number of cells recorded in parentheses. *P<0.05 and **P<0.01 as compared with control by ANOVA.

The typical time- and voltage-dependent inward currents evoked by increasing depolarizations from a holding potential of −40 mV were shown in the left panel of Fig. 2. Whole-cell Ca_L_ currents of cerebral VSMCs in SUS-28 d rats showed larger inward components of trace as compared with that recorded in CON-28 d (the left panel of Fig. 2A). In contrast, whole-cell Ca_L_ currents of small mesenteric VSMCs in SUS-28 d rats showed smaller inward components of trace as compared with that in CON-28 d rats (the left panel of Fig. 2B). The mean current-voltage relationship (*I–V*) curves which were further expressed in terms of current densities were shown in the right panel of Fig. 2. The *I–V* curves further showed that simulated microgravity significantly increased the Ca_L_ current densities in cerebral VSMCs (the right panel of Fig. 2A) and significantly decreases the Ca_L_ current densities in small mesenteric VSMCs (the right panel of Fig. 2B), respectively. However, 3-day recovery after removal of suspension could completely restore the Ca_L_ current densities of cerebral VSMCs to their control levels (the right panel of Fig. 2A) and partially recover the Ca_L_ current densities in small mesenteric VSMCs (the right panel of Fig. 2B). Only 7-day recovery after removal of suspension could completely restore the Ca_L_ current densities of small mesenteric VSMCs to their control levels (Fig. 2C). These results suggested there are a differential regulation and recovery of Ca_L_ current densities in cerebral and small mesenteric VSMCs, respectively.

### Simulated microgravity enhanced the expressions of Ca_L_ α_1C_-subunit in cerebral arteries and reduced the expressions of Ca_L_ α_1C_-subunit in small mesenteric arteries

**Figure 3:**
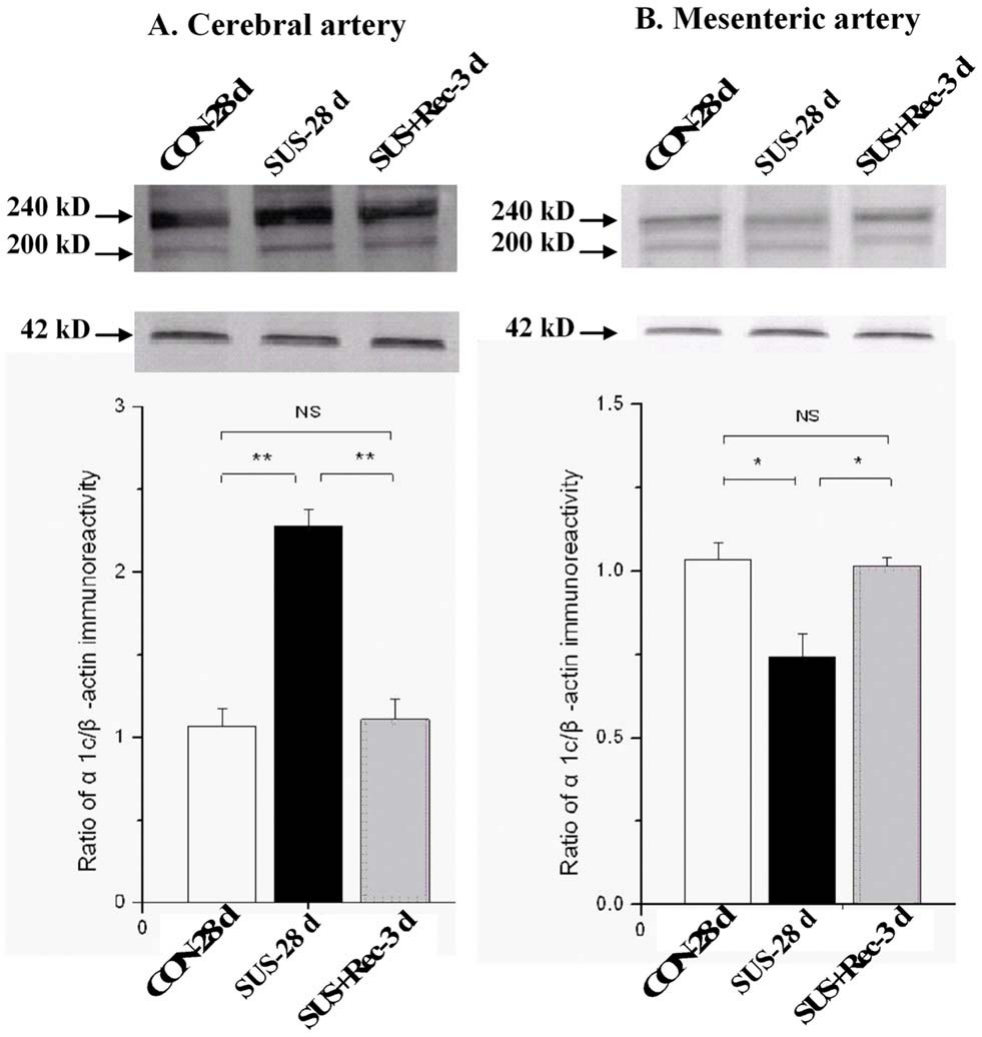
Comparison of Ca_L_ α_1C_-subunit expression by Western blotting in cerebral (A) and small mesenteric arteries (B) isolated from CON-28 d, SUS-28 d, and SUS+Rec-3 d rats. CON-28 d: 28-day simultaneous control rats, SUS: 28-day tail-suspended rats, SUS+Rec-3 d: tail-suspended rats submitted to a recovery period of 3 days at the end of 28-day suspension. Values are expressed as means ± SE from 4 independent experiments. **P<0.01 and *P<0.05 *vs.* CON by t-test.

The apparent masses of 200 and 240 kD doublet bands were shown in the membrane among CON-28 d, SUS-28 d, and SUS+Rec-3 d rats, which correspond to the predicted sizes of the short and long (or full length) forms of the Ca_L_ α_1C_-subunit protein in the cerebral and small mesenteric arteries, respectively (Fig. 3). As the internal control, the expressions of β-actin (42 kDa) were similar in different lanes in the bottom membrane, showing equal loading of proteins. The averaged data were expressed as percentage of the β-actin signal. As compared with that of CON-28 d rats, Ca_L_ channel α_1C_-subunit expressions of SUS-28 d rats increased by ∼105% in cerebral arteries and decrease by ∼32% in small mesenteric arteries, respectively. However, there were no significant differences in Ca_L_ channel α_1C_-subunit expressions of cerebral and mesenteric arteries between SUS+Rec-3 d and CON-28 d rats, which indicated that 3-day recovery could completely restore the Ca_L_ channel α_1C_-subunit expression in cerebral and small mesenteric arteries to their control levels, respectively. These results suggested that simulated microgravity differentially regulated the expression of Ca_L_ channels in cerebral and small mesenteric arteries, respectively. Furthermore, 3-day recovery can completely recover the effects of simulated microgravity on Ca_L_ channel expression in cerebral and small mesenteric arteries, respectively.

### Simulated microgravity increased the maximal increases of Ca^2+^ in cerebral VSMCs and decreased the maximal increases of Ca^2+^ in small mesenteric VSMCs in response to caffeine

**Figure 4:**
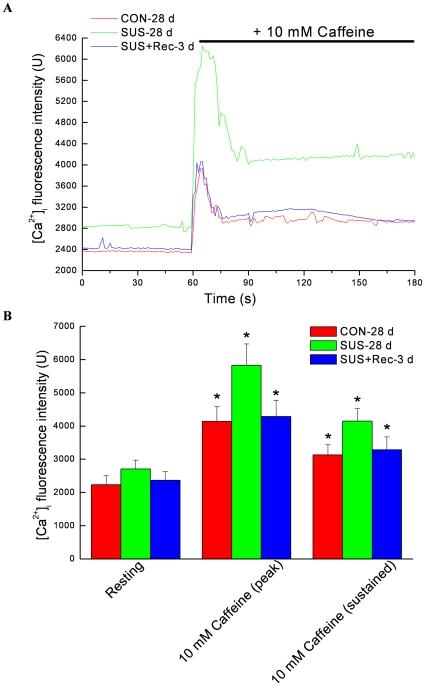
Caffeine elicits the increases of intracellular Ca^2+^ fluorescence intensity in cerebral VSMCs isolated from CON-28 d, SUS-28 d, and SUS+Rec-3 d rats. (A) Representative dot graph of Fluo-3/AM fluorescence recorded showed the typical transient increases of intracellular Ca^2+^ fluorescence intensity evoked by 10 mM caffeine. Caffeine was present at times shown by the horizontal bar. (B) Summarized data indicated the average changes of the intracellular Ca^2+^ fluorescence intensity before and during the application of caffeine. Values are means ± SE with the number of cells recorded in parentheses. CON-28 d: 28-day simultaneous control rats, SUS-28 d: 28-day tail-suspended rats, SUS+Rec-3 d: tail-suspended rats submitted to a recovery period of 3 days at the end of 28-day suspension. *P<0.05 as compared with their controls by t-test, respectively.

**Figure 5:**
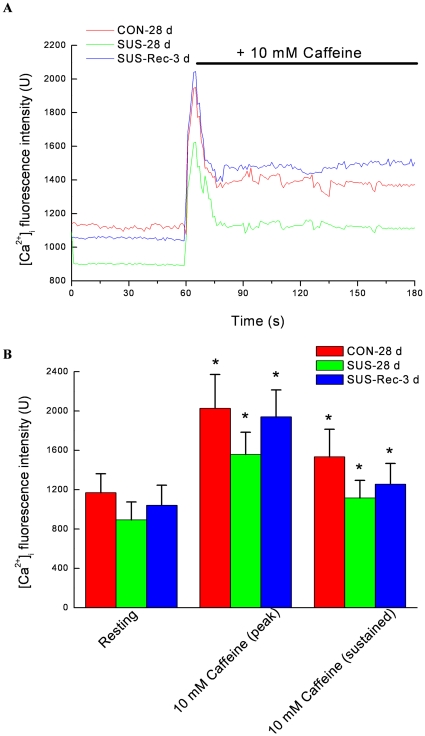
Caffeine elicits the increases of intracellular Ca^2+^ fluorescence intensity in small mesenteric VSMCs isolated from CON-28 d, SUS-28 d, and SUS+Rec-3 d rats. (A) Representative dot graph of Fluo-3/AM fluorescence recorded showed the typical transient increases of intracellular Ca^2+^ fluorescence intensity evoked by 10 mM caffeine. Caffeine was present at times shown by the horizontal bar. (B) Summarized data indicated the average changes of the intracellular Ca^2+^ fluorescence intensity before and during the application of caffeine. Values are means ± SE with the number of cells recorded in parentheses. CON-28 d: 28-day simultaneous control rats, SUS-28 d: 28-day tail-suspended rats, SUS+Rec-3 d: tail-suspended rats submitted to a recovery period of 3 days at the end of 28-day suspension. *P<0.05 as compared with their controls by t-test, respectively.

**Figure 6:**
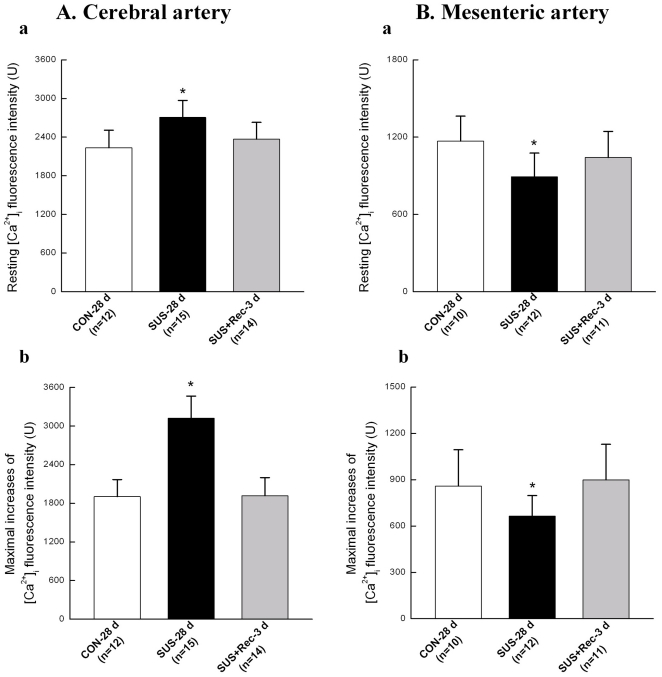
Comparison of resting level of Ca^2+^ fluorescence intensity and maximal increases of Ca^2+^ fluorescence intensity in cerebral (A) and small mesenteric VSMCs (B) isolated from CON-28 d, SUS-28 d, and SUS+Rec-3 d rats. Values are means ± SE with the number of cells recorded in parentheses. CON: 28-day simultaneous control rats, SUS: 28-day tail-suspended rats, SUS+Rec-3 d: tail-suspended rats submitted to a recovery period of 3 days at the end of 28-day suspension. *P<0.05 as compared with control by t-test.

The present data showed that a high concentration of caffeine (10 mM) evoked a transient peak increase of [Ca^2+^]_i_ and was followed by a sustained increase in [Ca^2+^]_i_ that was above the basal values in the continued presence of caffeine (Fig. 4 and Fig. 5). In present study, caffeine-evoked increases of [Ca^2+^]_i_ depended essentially on Ca^2+^ release from the RyRs in SR because the Ca^2+^-free solution (removal of Ca^2+^ from extracellular solution) was used to diminish Ca^2+^ influx through voltage-dependent Ca^2+^ channels in plasma membrane. As compared with that of CON-28 d rats, 28-day simulated microgravity significantly induced a higher resting [Ca^2+^]_i_ level in cerebral VSMCs (Fig. 6Aa) and a lower resting [Ca^2+^]_i_ level in small mesenteric VSMCs (Fig. 6Ba), respectively, which were consistent with previous report [16], [18]. However, 3-day recovery after removal of suspension could restore the resting [Ca^2+^]_i_ to their control level in cerebral and small mesenteric VSMCs, respectively (Fig. 6Aa and Fig. 6Ba). In addition, the acute applications of 10 mM caffeine evoked a significant augment of the maximal increase of [Ca^2+^]_i_ by ∼64.0% in cerebral VSMCs isolated from SUS-28 d rats as compared with that of CON-28 d. However, the maximal caffeine-induced increase of [Ca^2+^]_i_ in SUS+Rec-3 d rats was similar to that obtained in CON-28 d rats, indicating a complete recovery of ryanodine-sensitive Ca^2+^ releases in cerebral VSMCs (Fig. 4 and Fig. 6Ab). In contrast, prolonged suspension resulted in a significant reduction of the maximal caffeine-induced increase of [Ca^2+^]_i_ by ∼22.7% in small mesenteric VSMCs as compared with that of CON-28 d rats. Correspondly, there were no significant differences in the maximal caffeine-induced increase of [Ca^2+^]_i_ between SUS+Rec-3 d and CON-28 d rats, suggesting a complete recovery of ryanodine-sensitive Ca^2+^ releases in small mesenteric VSMCs (Fig. 5 and Fig. 6Bb). These results suggested that simulated microgravity differentially regulated the function of ryanodine-sensitive Ca^2+^-releases in cerebral and small mesenteric arteries, respectively. Furthermore, 3-day recovery can completely recover the effects of simulated microgravity on ryanodine-sensitive Ca^2+^ releases in cerebral and small mesenteric arteries, respectively.

## Discussion

The principal and novel finding of this work is that simulated microgravity differentially regulates the intracellular Ca^2+^ by its effects on Ca_L_ channels in plasma membrane and the ryanodine-sensitive Ca^2+^ releases from SR, i.e., simulated microgravity increased the functions of Ca_L_ channels and ryanodine-sensitive Ca^2+^ releases in cerebral VSMCs, whereas, simulated microgravity decreased the functions of Ca_L_ channels and ryanodine-sensitive Ca^2+^ releases in small mesenteric VSMCs. In addition, 3- or 7-day recovery after removal of suspension could restore the functions of Ca_L_ channels and ryanodine-sensitive Ca^2+^ releases to their control levels in cerebral and small mesenteric VSMCs, respectively. These differential regulations of Ca_L_ channels and ryanodine-sensitive Ca^2+^ releases in cerebral and small mesenteric VSMCs may be responsible for the altered autoregulation of cerebral vasculature and the inability to adequately elevate peripheral vascular resistance.

### Differential adaptations in cerebral and small mesenteric arteries during real/simulated microgravity

There is a blood pressure gradient from the head to the feet in humans at 1 G in the upright posture. When exposed to microgravity, all gravitational blood pressure gradients are lost. Thus, blood vessels in cerebral circulation are exposed to higher than normal 1-G blood pressure, whereas vessels in lower body regions are exposed to lower than normal upright 1-G blood pressure. Therefore, the differential adaptations in cerebral and hindquarter arteries during real/simulated microgravity have been postulated to be a basical process of vascular autoregulation in response to sustained elevation or reduction in local transmural pressures, which may contribute to cardiovascular dysfunction and ultimately lead to the post spaceflight orthostatic intolerance [3], [7]. In addition, after release from simulated microgravity, the functional and structural adaptations in the forebody arteries or hindquarter arteries of rats were partially restored (7-day recovery) and fully recovered (35-day recovery) to their control levels, which means that these changes were reversible within the time frame of previous experiments [7].

### Differential regulation of Ca_L_ channels in cerebral and small mesenteric arteries of simulated microgravity rats

Ca_L_ channels of the Cav1.2 gene family are the principal Ca^2+^ influx pathway in VSMCs and participate in multiple physiological functions, including excitation-contraction coupling, cell apoptosis, and cell proliferation in small resistance vessels. Ca_L_ channels are heteromeric complex composed of a central pore-forming α_1C_ subunit and regulatory β, γ, and α_2δ_ subunits in VSMCs. The pore-forming α_1C_ subunit (about 190–240 kDa) is considered to confer most functional properties to the Ca_L_ channel. It is currently believed that membrane depolarization of VSMCs associated with elevated blood pressure permits extracellular Ca^2+^ entry through Ca_L_ channels, whereas, membrane hyperpolarization of VSMCs associated with lower blood pressure inhibits extracellular Ca^2+^ entry through Ca_L_ channels [12], [25]. Therefore, Ca_L_ channels regulate a tonic level of vascular tone and contribute to the dynamic autoregulation in the vascular beds. The abnormalities of Ca_L_ channels are regarded as part of the extensive biological and morphological adaptations during the pathogenesis of vascular diseases. By whole-cell and Western blotting, the present work demonstrated that simulated microgravity depolarized cerebral VSMCs (Fig. 1A) and then upregulated the function of Ca_L_ channels in cerebral arteries of rats (Fig. 2A and Fig. 3A), which are in agreement with the reports that hypertensive rats associated with anomalous arterial tone show an increased function of Ca_L_ channels to sustain the elevated Ca^2+^ influx [12]. In contrast, the present work also indicated that simulated microgravity hyperpolarized small mesenteric VSMCs (Fig. 1B) and then downregulated the function of Ca_L_ channels in small mesenteric arteries of rats (Fig. 2B and Fig. 3B), which were in consistent with the reports that inhibition of Ca_L_ channels due to hypotension in shock rats [26]. In addition, the membrane potential (Fig. 1) and the function of Ca_L_ channels (Fig. 2 and Fig. 3) could return to their control levels in cerebral and small mesenteric arteries after 3- or 7-day recovery after removal of suspension, which indicated that these differential changes are reversible when the simulated microgravity were released.

### Differential regulation of ryanodine-sensitive Ca^2+^-releases in cerebral and small mesenteric arteries of simulated microgravity rats

RyRs are tetrameric channel proteins which are located in the SR plasma membrane. Three isoforms of RyRs have been identified as RyR1, RyR2, and RyR3, which are all present in smooth muscle cells [27]. RyRs are one of the main actors in the generation of Ca^2+^ signals, transient local releases of Ca^2+^, and this Ca^2+^ events provides the primary pathway for SR Ca^2+^ release into the cytosol underlying the excitation-contraction coupling. There is an indirect coupling between Ca_L_ channels in the plasma membrane and RyRs in the SR of arterial VSMCs, i.e., RyRs can be opened by elevations in cytosolic Ca^2+^ by opening Ca_L_ channels, termed Ca^2+^-induced Ca^2+^ release (CICR), and thus can contribute to the overall rise in the concentration of intracellular Ca^2+^ [13], [28]. Caffeine is commonly used to activate RyRs by increasing the affinity of the Ca^2+^ activator site for Ca^2+^, whereas ruthenium red is a frequently used inhibitor. At concentrations of >5 mM, Caffeine activates all RyR isoforms to cause Ca^2+^ release from the SR [27]. The massive release of Ca^2+^ in response to high mM concentrations of caffeine reflects the amount of calcium within the SR. Structural and functional studies indicate the important role of the SR and any alteration of ryanodine-sensitive Ca^2+^-releases from the SR could consequently alter contractile responsiveness of arterial smooth muscle cells in cardiovascular diseases [29]. The present study demonstrated that 28-day simulated microgravity enhanced the function of ryanodine-sensitive Ca^2+^ releases (the maximal increases of caffeine-induced [Ca^2+^]_i_ transients) in cerebral VSMCs (Fig. 4 and Fig. 6Ab) and reduced the function of ryanodine-sensitive Ca^2+^ releases (the maximal increases of caffeine-induced [Ca^2+^]_i_ transients) in small mesenteric VSMCs (Fig. 5 and Fig. 6Bb). However, 3-day recovery after removal of suspension could completely recovery the function of ryanodine-sensitive Ca^2+^-releases in cerebral and small mesenteric VSMCs, respectively (Fig. 6), which also suggested that the changes of ryanodine-sensitive Ca^2+^-releases due to simulated microgravity are reversible when the simulated microgravity were released.

### Practical implications of differential regulation of intracellular Ca^2+^ in cerebral and small mesenteric VSMCs during simulated microgravity

Vascular contraction is dependent on an increase in intracellular free Ca^2+^ concentration as a result of rapid Ca^2+^ release from intracellular stores, chiefly SR, and from Ca^2+^ influx via plasma membrane Ca_L_ channels. Therefore, the differential regulation of the intracellular Ca^2+^ in cerebral and small mesenteric VSMCs of simulated microgravity rats might be an underlying mechanism of microgravity-induced orthostatic intolerance. For cerebral vessels, the upregulation of Ca_L_ channels (Fig. 2 and Fig. 3) accompanied with the enhanced function of ryanodine-sensitive Ca^2+^-releases (Fig. 4 and Fig. 6) may resulted in an increased concentration of intracellular Ca^2+^ (Fig. 6) for the maintenance of an elevated myogenic ton. The elevated Ca^2+^-dependent vascular tone is an important protective mechanism against an increased cerebral perfusion pressure induced by simulated microgravity to reduce the risk of excessive capillary filtration, cerebral edema and possible stroke [6], [17]. In contrast, splanchnic and muscular vascular beds are the main contributors to the maintenance of peripheral vascular resistance, so the downregulation of Ca_L_ channels (Fig. 2 and Fig. 3) accompanied with the reduced function of ryanodine-sensitive Ca^2+^-releases (Fig. 5 and Fig. 6) in small mesenteric arteries may lead to a decreased concentration of intracellular Ca^2+^ (Fig. 6) for the maintenance of an attenuated myogenic tone. The attenuated Ca^2+^-dependent vascular tone is also an important protective mechanism against a reduced mesenteric perfusion pressure induced by simulated microgravity [6], [17]. However, the altered Ca^2+^-dependent vascular tone might be an important aspect responsible for the altered autoregulation of cerebral vasculature and the inability to adequately elevate peripheral vascular resistance during postspaceflight orthostatic intolerance. It is supposed that the differential adaptations in the functions of Ca_L_ channels and ryanodine-sensitive Ca^2+^ releases are an immediate and early adaptive response to microgravity, which are reversible and could recover when the microgravity exposure disappears.

### Limitations of the study

First, the present work showed that 3-day recovery after removal of suspension could completely restore the effects of simulated microgravity on the function of ryanodine-sensitive Ca^2+^ releases from SR in cerebral and small mesenteric VSMCs of rats (Fig. 6). However, 3-day recovery after removal of suspension could completely restore the function of Ca_L_ channels in cerebral VSMCs (Fig. 2A) and partly restore the function of Ca_L_ channels in small mesenteric VSMCs (Fig. 2B). Only 7-day recovery after removal of suspension could completely restore the function of Ca_L_ channels in small mesenteric VSMCs (Fig. 2C). What is the reason for the differential recovery in Ca_L_ channels of cerebral and small mesenteric VSMCs isolated from simulated microgravity rats needs the further investigation. Second, it is notable that two families of Ca^2+^ release channels exist in the SR, the inositol 1,4,5-trisphosphate receptors (IP_3_Rs) and the RyRs. In the present study, the increased Ca^2+^ transients were mainly due to activate RyRs because caffeine may either direct or indirect inhibition of IP_3_Rs [23]. However, activation of IP_3_Rs has been also reported to evoke localized Ca^2+^ transients and contribute to the global elevation of intracellular Ca^2+^ [30], so it is very interesting to study the role of IP_3_Rs during simulated microgravity in the further work.

### Conclusion

Taken together, the present work showed that simulated microgravity differentially regulated the intracellular Ca^2+^ determined by its effects on the functions of Ca_L_ channels and ryanodine-sensitive Ca^2+^ releases in cerebral and mesenteric VSMCs of rats, respectively. However, 3- or 7-day recovery after removal of simulated microgravity could restore these functions to their control levels in cerebral and mesenteric VSMCs, respectively. Our results suggested that differential regulation of intracellular Ca^2+^ might be the initiating factors leading to the functional and structural adaptations in the vessel caused by altered distribution of pressures and flows within the vasculature during microgravity.
